# A Review of Three-Dimensional Medical Image Visualization

**DOI:** 10.34133/2022/9840519

**Published:** 2022-04-05

**Authors:** Liang Zhou, Mengjie Fan, Charles Hansen, Chris R. Johnson, Daniel Weiskopf

**Affiliations:** ^1^National Institute of Health Data Science, Peking University, Beijing, China; ^2^Scientific Computing and Imaging Institute, University of Utah, Salt Lake City, USA; ^3^Visualization Research Center (VISUS), University of Stuttgart, Stuttgart, Germany

## Abstract

*Importance*. Medical images are essential for modern medicine and an important research subject in visualization. However, medical experts are often not aware of the many advanced three-dimensional (3D) medical image visualization techniques that could increase their capabilities in data analysis and assist the decision-making process for specific medical problems. Our paper provides a review of 3D visualization techniques for medical images, intending to bridge the gap between medical experts and visualization researchers.*Highlights*. Fundamental visualization techniques are revisited for various medical imaging modalities, from computational tomography to diffusion tensor imaging, featuring techniques that enhance spatial perception, which is critical for medical practices. The state-of-the-art of medical visualization is reviewed based on a procedure-oriented classification of medical problems for studies of individuals and populations. This paper summarizes free software tools for different modalities of medical images designed for various purposes, including visualization, analysis, and segmentation, and it provides respective Internet links.*Conclusions*. Visualization techniques are a useful tool for medical experts to tackle specific medical problems in their daily work. Our review provides a quick reference to such techniques given the medical problem and modalities of associated medical images. We summarize fundamental techniques and readily available visualization tools to help medical experts to better understand and utilize medical imaging data. This paper could contribute to the joint effort of the medical and visualization communities to advance precision medicine.

## 1. Introduction

In recent years, with advances in computing power and algorithms, the impact of technology in medicine is greater than ever and keeps increasing. Strategic plans focusing on promoting the application and development of medical technologies have been made worldwide. In 2018, the General Office of the State Council of China issued “Opinions on Promoting the Development of ‘Internet Plus Healthcare,’” calling for strengthening the integration, sharing, and application of clinical and research data and supporting the research and development of health-related artificial intelligence (AI) technology, medical robots, etc. (http://www.gov.cn/zhengce/content/2018-04/28/content_5286645.htm). A three-year action plan was released to develop AI, which puts a priority on expanding the clinical application such as medical image-assisted diagnosis systems (http://www.cac.gov.cn/2017-12/15/c_1122114520.htm). In the same year, the National Institutes of Health (NIH), USA, released the NIH Strategic Plan for Data Science, which proposed that technological innovations such as machine learning, deep learning, AI, and virtual reality (VR) could revolutionize biomedical research over the next 10 years (https://datascience.nih.gov/nih-strategic-plan-data-science).

Medical images, such as computerized tomography (CT), magnetic resonance imaging (MRI), and diffusion tensor imaging (DTI), are the backbone of modern medical practices and research. Furthermore, medical images are a core data source and target for analysis in AI and VR in aforementioned strategic plans. Therefore, the analysis and understanding of medical images are of utmost importance in medical technologies. In practice, most if not everyone in health science areas is familiar with medical images, and many use medical images in daily work. Medical images are massive and complex and hard to explore and gain insights with traditional statistical methods that do not involve the expertise of humans. Therefore, technologies that have humans in the loop are needed for medical image exploration and analysis, and medical experts could make the best of the potential values of medical images to enable high-quality healthcare solutions. Vision is known to be the major and most efficient perception mechanism for humans. Visualization transforms data into interactive visual representations to facilitate data understanding and exploration through visual perception and human-computer interaction, and it can present medical images in 3D with high accuracy. Visualization integrates the cognitive advantage of humans and the computational advantage of computers for data mining [1] and decision-making [2] and is an effective data analysis technology for medical images. Throughout this paper, we understand the term medical image visualization as 3D visualization of medical images.

We first report on the scope and sources of papers involved (Section 2). We then summarize fundamental techniques that enable the visualization of various types of medical images (Section 3). Next, in Section 4, specialized medical imaging visualization methods are reviewed based on our medical procedure-oriented and scale-based taxonomy. In Section 5, we summarize visualization techniques that may have potential medical applications and discuss limitations of medical image visualization. Finally, we list free software tools that are readily available online for medical image visualization (Section 6) and conclude the overview in Section 7.

## 2. Scope

Medical visualization, in general, is systematically covered in the textbook by Preim and Bartz [3]. In this paper, we focus on techniques of 3D medical image visualization and specific techniques for various medical problems based on imaging categorized by the medical procedure and the scale of studies.

There are several reviews of visualization techniques for medical images [4– 8]. However, the target audience of these reviews is visualization researchers rather than medical experts. These technical-oriented reviews offer in-depth discussions on visualization techniques for specific medical data types with fine-grained taxonomy, including perceptually motivated 3D medical image data visualization [4], multimodal medical data [6], medical flow data [8], cardiac 4D data [5], and flattening-based medical visualization [7].

Our paper, instead, is to provide medical experts with a general overview of this highly relevant field. We believe that our review achieves a balance between the coverage of techniques and the relevance for medical experts, and our goal is to convey existing visualization techniques that are potentially useful for health care experts in their research and clinical practices.

We focus on techniques that generate 3D visualizations of medical images. While other representations of 3D medical images exist, e.g., 2D representations with flattening visualization techniques, we do not include them in our review and readers are referred to a survey elsewhere [7]. Our review includes classic papers for fundamental techniques (Section 3) and visualization papers from mainstream visualization venues, e.g., IEEE Transactions on Visualization and Graphics (TVCG), Computer Graphics Forum (CGF), the IEEE VIS conference, the EuroVis conference, the IEEE PacificVis conference, and Computers and Graphics. Specifically, with a few exceptions, papers covered in Section 4 were selected by first searching for the term “medical visualization” on the two major venues—IEEE TVCG (including the VIS conference) and CGF (including the EuroVis conference)—with the publication time within the last 15 years (2006–2021), and then, we carefully examined each paper found in the search and included papers with direct relevance to medicine and 3D visualization of medical images.

Unlike a previous review on biomedical visualization techniques [9], our overview covers advanced visual analysis methods for medical images that are arranged based on a taxonomy of medical problems from individuals to populations (Section 4) and also includes advances in fundamental visualization methods (Section 3). This paper also features an overview of techniques that could improve spatial perception, which is vital for the understanding of medical images in 3D. Overall, rather than a comprehensive review of the literature on the topic of medical image visualization, this review provides a big picture of the subject of study and introduces state-of-the-art techniques driven by specific medical problems.

## 3. Fundamental Medical Image Visualization Techniques

We understand medical images as data over 3D spatial domains that have values defined for each point in space and possibly in time (e.g., time-varying data), namely, *fields*. Medical imaging data can be abstracted as fields where data values are defined everywhere within the spatial domain, for example, fluid characteristics of tissues in an MR image.

In practice, the field data is sampled and stored as discrete data points in images of various formats, e.g., DICOM (Digital Imaging and Communications in Medicine), NII (NIfTI-1 data format), TIFF (Tag Image File Format), and RAW (Raw image). We denote a data point v of a medical image data as

(1)v:x,y,z,t,s1,s2,⋯,sm,

where x,y,z are the spatial coordinates, t is the time, and $s_{1},s_{2},\cdots ,s_{m}$ are physical attributes of this data point, for example, the Hounsfield scale (for CT) or T1- (longitudinal relaxation time) weighted, T2- (transverse relaxation time) weighted, and FLAIR-fluid-attenuated inversion recovery, and m denotes the number of attributes.

**Figure 1:**
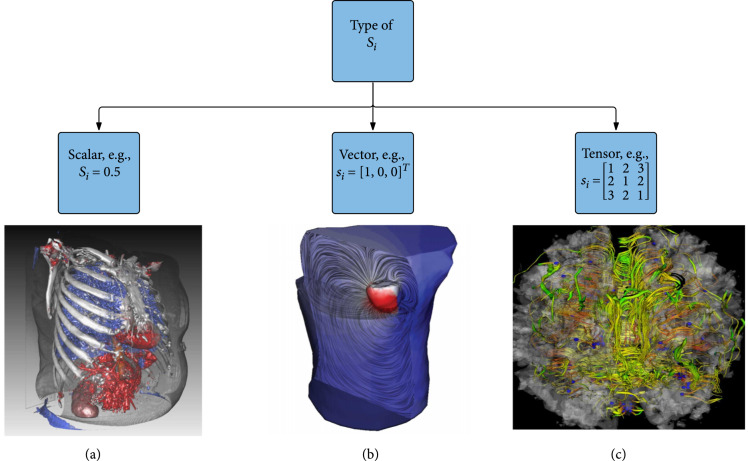
Classification of fundamental visualization techniques for different types of medical image data. Scalar, vector, and tensor image data visualization is discussed in this order in the remainder of this section. Example visualizations include a direct volume rendering of a CT scan of a torso [10] for scalar data, a streamline visualization of a bioelectric field simulation of a torso for vector data, and a tube-based tractography visualization of a brain [11] for tensor data. Reprinted from Computers & Graphics, Vol. 36, No. 6, Zhou and Hansen [10], Transfer Function Combinations, 596-606, Copyright 2012, with permission from Elsevier (a). Reprinted, with permission, from the SCI Institute (b). ©2007 SPIE. Reprinted, with permission, from Weldeselassie et al. [11] (c).

Medical image data can be classified into three types: *scalar*, *vector*, and *tensor*, based on the mathematical properties of physical attributes s. When m>1, the medical image data are multifield data that can be a mixture of various data types, e.g., scalars and vectors or scalars and tensors, that are typical in real medical practices. Advanced techniques for multifield visualization are covered in Section 4. Figure 1 shows the classification of visualization techniques for medical images with examples (Figures 1(a)– 1(c)) of a scalar CT scan of a torso, a flow simulation of a torso, and a tensor field visualization of a brain. In the remainder of this section, we focus on the case of single-typed data, i.e., each attribute of s has the same type, and discuss fundamental visualization techniques for each type of data.

### 3.1. Scalar Image Visualization

If each attribute of s of equation (1) is a scalar, i.e., a quantity without direction, the data is scalar volumetric data in the context of 3D medical imaging. In general, volume visualization techniques can be categorized into two classes: indirect volume visualization and direct volume visualization. Indirect volume visualization extracts surfaces with certain data values to *indirectly* visualize a volumetric data with surface meshes. Since the extracted surfaces there have the same data value, for example, parts of the skin that have a specific Hounsfield scale in a CT scan, such methods are also called isosurface rendering. In contrast, direct volume visualization or volume rendering *directly* visualizes the volume data without extracting any surfaces and allows the “see-through” of internal structures of a medical image, for example, the brain within a head MR scan. 

Isosurface rendering is usually realized with the marching cubes method [12]. The marching cubes algorithm traverses data cells, i.e., cubes, through the data volume and uses a lookup table to efficiently determine the topology of the isosurface and computes the intersection of the isosurface and the cell to construct triangles accordingly. The triangle mesh is then visualized with a color palette specified by the user. Isosurfaces can be generalized to “thick” interval volumes and even be combined with direct volume rendering in a unified framework [13]. However, indirect volume rendering shows only a limited number of features as surfaces, and it does not provide the volumetric look within medical images. 

#### 3.1.1. Direct Volume Rendering

For a systematic introduction to direct volume rendering, we refer the reader to the course notes [14] or the book on real-time volume graphics [15]. Volume rendering is based on optics, and all techniques are approximations to the solution of the light propagation equation [16] in volumetric materials. Later, volume rendering was extended to include medical images—initially focusing on CT human head scans—along with surface shading [17,18].

**Figure 2:**
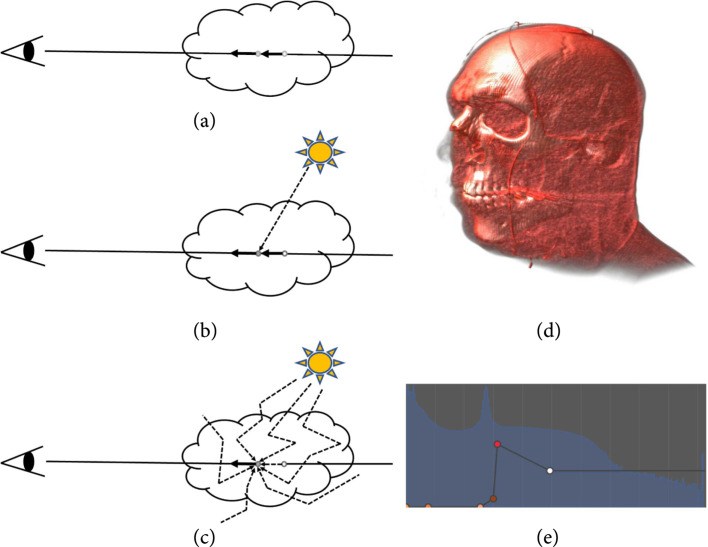
Optical models of direct volume rendering: (a) emission and absorption, (b) local illumination, and (c) global illumination. A CT human head scan is visualized in (d) using local illumination with (e) a 1D transfer function.

An optical model determines how particles in the media interact with light [19]. We illustrate the direct volume rendering problem with typical optical models in Figure 2: the eye symbol represents the viewer, the cloud indicates the scalar volumetric data, a ray (the straight line) is shot from the viewer through the volume, the current volume sample is indicated by a gray dot, and the light source is drawn as the sun. The final color of the ray “seen” by the viewer is determined by solving the volume rendering integral with a given optical model. Particles emit and absorb light in the emission and absorption model (Figure 2(a)), and the volume sample only receives lights from samples to its back, e.g., the white dot, along the ray. On top of the emission and absorption model, a local illumination model adds local reflections of light from the light source. Here, light from the source along the straight line to the volume sample may be attenuated as illustrated in Figure 2(b). Alternatively, attenuation between the light source and the point in the volume might be neglected. Finally, global illumination (Figure 2(c)) considers a full model of scattering that leads to complex light paths and interactions, as indicated by the irregular polylines. Currently, most applications rely on local illumination (often, without attenuation from the light source to the point of local illumination) as a compromise between rendering quality and computational cost. In contrast, the global illumination model can achieve photorealistic visual effects, such as shadows and translucency, that improve spatial perception, which is important for medicine as discussed in Section 3.1.3.

Volume rendering, even with a simple optical model, has high computational cost. Due to the limitation of computer hardware, interactivity was not achieved even with hardware acceleration [20] in early work. Therefore, practical use of direct volume rendering of medical images was not feasible back then.

With the advent of graphics processing units (GPUs), interactive volume rendering techniques were devised [21– 23]. Among them, the GPU-based ray casting [23] method is currently the standard volume rendering technique in most applications. The main advantages of GPU-based ray casting lie in that no geometry has to be generated, and the implementation is straightforward. Interactive volume rendering is also available on mobile devices [24] that can facilitate ubiquitous visualization and analysis of medical images.

#### 3.1.2. Visual Data Exploration with Transfer Functions

The medical image data and its rendering are linked by a *transfer function*—a mapping from the data value to optical properties, i.e., color and opacity of the volume. The idea is already realized in early volume rendering papers [17,18], but they did not use transfer functions as a data exploration tool. However, for medical visualization, transfer functions are the main means of interactive visual mining/exploration of scalar medical images. A survey of transfer functions for volume rendering can be found elsewhere [25].

The basic and most frequently used transfer functions are one-dimensional (1D) transfer functions that typically map data values of the scalar volume, i.e., the grayscale value of images, to color and opacity. An example of volume rendering of a CT head scan is shown in Figure 2 with a screenshot of a 1D transfer function widget (Figure 2(e)). The distribution of data values is shown in blue in the background, and the transfer function is shown in the foreground: the line in black indicates the opacity, and the color is set by linear interpolation of the colored dots. Here, the skin and muscles are set to yellow and brown of low opacity, blood vessels are assigned red with high opacity, and bones are set to white of high opacity. Although relatively easy to use, 1D transfer functions have limited feature classification capability; for example, they cannot clearly separate the bone and the blood vessels as shown in Figure 2(d), due to the partial-volume effect of medical imaging. A preintegrated technique can be employed to improve the quality of volume rendering with 1D transfer functions [26].

Artifacts caused by the partial-volume effect cannot be resolved by using only the single scalar image data value. Therefore, more attributes are measured as in the multimodal medical imaging, e.g., T1, T2, and FLAIR channels in MRI, or derived from the original image data. Accordingly, multidimensional transfer functions use the attributes jointly for improved classifications. Two-dimensional transfer functions that enhance boundaries of volumes are the typical choice on most occasions as the second attribute is easily derived from the original data [27,28]. Interaction widgets for 2D transfer functions [28] have become standard in most of the current visualization tools described in Section 6.

Designing multidimensional transfer functions for more than two attributes is challenging. One common approach is to explore the value domain of multimodal medical images. Several techniques [29– 31] based on multidimensional diagrams facilitate selections of specific value ranges of each data attribute. However, value domain approaches are unfamiliar to medical experts. In contrast, the spatial domain, e.g., on the slices of images, is preferred. Accordingly, slice-based multidimensional transfer function methods are available. For example, a machine learning-based method allows the user to draw directly on slices for training [32]. Another example is slice-based sketching combined with parallel coordinates and scatterplots for multimodal medical images [33]. A semiautomatic method sets transfer functions through drawing features of interest on slices with probabilistic boundary lassos and approximate optimization [34].

#### 3.1.3. Techniques for Improved Spatial Perception

Although local illumination is widely used in volume rendering, more realistic rendering is often necessary for clinical purposes as it provides important depth cues allowing accurate spatial perception. Therefore, global illumination has been a core research topic in volume rendering. A general and comprehensive global illumination model that creates various visual phenomenon is available in computer graphics [37].

**Figure 3:**
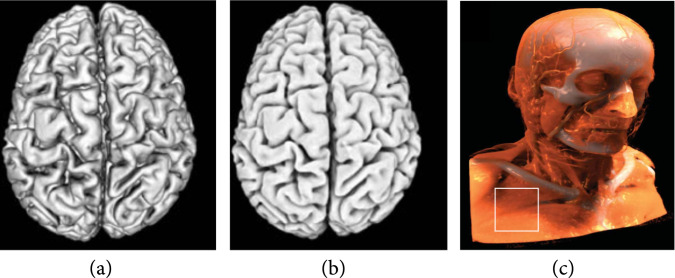
Direct volume rendering of a brain scan using (a) the traditional local illumination model and with (b) the directional occlusion shading [35] that approximates global illumination to enhance depth perception. ©2009 John Wiley & Sons, Inc. Reprinted, with permission, from Schott et al. [35]. A more advanced global illumination technique [36] generates the photorealistic rendering of a CT scan in (c). ©2014 IEEE. Reprinted, with permission, from Ament et al. [36].

In the case of direct volume rendering, Monte Carlo sampling enables global illumination [38]; however, the gradual rendering from a coarse to fine visualization and the noisy look makes its integration to the clinical pipeline premature. Shadows and translucency are enabled by half-angle slicing [22], and directional occlusion is also available [35]. Figure 3(b) shows the volume rendering of an MRI brain scan with directional occlusion. Compared to Figure 3(a) with the traditional Phong illumination—a frequently used local illumination method—directional occlusion (Figure 3(b)) creates important depth cues that improve the perception of the complex creases of the brain.

Scattering and shadows are also made possible with GPU-based ray casting that achieves high image quality [39]. Advanced global illumination models [36,40] are available to include scattering and soft shadowing in ray-casting volume rendering. Figure 3(c) shows the volume rendering of a CT scan with a low-pass shadowing model with scattering that provides translucency. These methods could aid medical experts in their clinical work to quickly and accurately locate features of interest in the visualization, which is not possible with traditional local illumination models.

### 3.2. Vector Image Visualization

When attributes s (equation (1)) are vectors, the medical image to be visualized is a vector field. Vector field visualization is extensively used and studied in computational fluid dynamics for science and engineering, and a survey of general vector field visualization techniques is available [43]. In medicine, vector field data in terms of fluid flow from phase-contrast MR (PC-MR) scans are typically used for understanding pathological cerebral aneurysm haemodynamic, nasal aerodynamics, and aortic haemodynamics; bioelectric simulations based on electrocardiography (ECG) in cardiology and electroencephalography (EEG) or magnetoancephalography (MEG) in neurology are another source of vector image data [44]. 

For more details on medical vector field data visualization, we refer the reader to several surveys [5, 8, 45]. In this section, we briefly review medical vector field visualization techniques in the following order: *direct methods*, *geometry methods*, *feature methods*, and techniques for accurate spatial perception. Segmentation and mesh generation are also important processes in vector medical image visualization but are beyond of the scope of our review. 

#### 3.2.1. Direct Methods

Direct methods do not explicitly extract any geometry or features in a vector field. One strategy visualizes vector information with glyphs on 2D slices [46]. Another strategy converts vector information into multiple scalars and visualizes them with color coding in slices or volumes using direct volume rendering. However, direct methods do not visualize trajectories of particles in the vector field, i.e., global path features, which is one of the focal points of vector field analysis. Therefore, direct methods see limited use in vector medical image visualization.

#### 3.2.2. Geometry Methods

**Figure 4:**
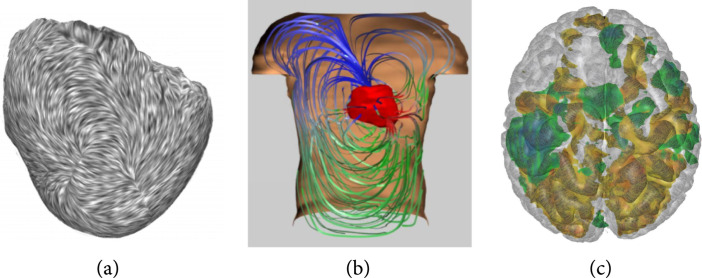
Vector field visualization of medical images. The LIC method is used to visualize (a) a simulation of the bioelectric field of the heart [41]. ©2008 IEEE. Reprinted, with permission, from Li et al. [41]. Streamlines are visualized as colored lines in bioelectric field simulations in (b) a torso (reprinted, with permission, from the SCI Institute). The visualization of a brain in (c) applies LIC with isosurfacing for an MRI scan along with volume rendering of a functional MRI (fMRI) [42]. ©2007 SPIE. Reprinted, with permission, from Schafhitzel et al. [42].

Extracting and visualizing representative geometry in the vector field, e.g., streamlines, pathlines, or streaklines, are effective and well-received methods. Geometry methods extract global path features from seeded points in the image [47]. Streamlines are visualized as 3D lines or tubes colored by the desired property, for example, the electrical potentials of electric fields of a torso in Figure 4(v) or a brain with the volume rendering as context in Figure 4(d). The analysis of flow patterns is aided with image slices [48] similar to the case of volume rendering, where a cutting plane is often used. The extraction of global path features is sensitive to seed points; a probing tool facilitates seeding in images [49]. However, automatic seeding remains as a challenging and open question.

Alternative to visualizing 3D geometry as lines, tubes, or surfaces, global path features can be shown with textures. Line integral convolution (LIC) [50] visualizes the vector field by convolving a noise image with a low-pass filter along streamlines extracted from the data image. The LIC method can be extended to curved surfaces [41,51], for example, on the surface of a heart [41] as shown in Figure 4(a) or a torso as shown in Figure 4(b).

#### 3.2.3. Feature Methods

Feature methods extract features of interests, e.g., sinks, sources, saddle points, vortices, and regions of abnormal flow velocity, in vector fields. We cover specialized methods for medical fluid flows; for other vector field data, we refer the readers to a general overview of texture-based vector field visualization [43]. Extracting vortex regions is a vital process in a number of vector medical image visualization techniques [52,53]. Vortex cores in blood vessels are related to blood transportation mechanisms in the aorta and the left ventricle [53]. Vortex regions are extracted for analysis of potential malfunctions [52].

Line predicates [54] are functions used for querying integral lines (streamlines or pathlines) that meet certain properties of interest. These functions are flexible and especially suitable for vector medical images as flow features such as velocity, shapes of lines, and distances to the vessel wall can be encoded. Blood with high residence times [55] and impingement zones of cerebral aneurysms [56] can be extracted with line predicates.

#### 3.2.4. Techniques for Improved Spatial Perception

Spatial perception of path geometry, lines or tubes, is difficult in 3D due to many factors, e.g., cluttering, inaccurate depth perception, occlusion, and inaccurate perception of orientation. Methods that improve spatial perception are available to complement aforementioned visualization techniques (Sections 3.2.1– 3.2.3). Particles in the flow field can be drawn as different shaped glyphs [52] or cartoon-styled stretched ellipsoids or short pathlines (pathlets) [49]. Drawing whole extracted lines as illuminated streamlines [57] or with halos [58,59] is another strategy for visualization with enhanced spatial perception.

**Figure 5:**
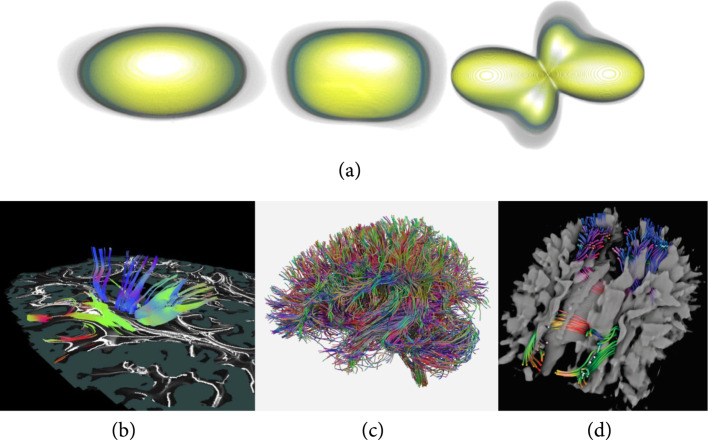
Visualizations of diffusion tensor medical images with (a) glyphs [63], (b) hybrid voxel-based and tractography method [64], and (c, d) tractography methods [61, 62]. Different uncertainty-aware glyph encodings are shown in (a), from left to right: ellipsoids, superquadrics, and fourth-order homogeneous polynomial [63]. A combined rendering of voxel-based visualization of characteristic curves along with extracted tractography is shown in (b) [64]. For tractography visualizations, extracted fiber tracts of the brain are visualized as tubes with shadows with (c) ray tracing [62] and are combined into the context of volume rendering of scalar MRI with unified volume and surface occlusion shading [61] (d). ©2012 IEEE. Reprinted, with permission, from Jiao et al. [63] (a). ©2011 IEEE. Reprinted, with permission from, Hlawatsch et al. [64] (b). ©2019 John Wiley & Son, Inc. Reprinted, with permission, from Han et al. [62] (c). ©2013 IEEE. Reprinted, with permission, from Schott et al. [61] (d).

Global illumination is superior to local illumination in user performance for locating and comparing features with tube/line renderings as shown in a comparative study [60]. Shadows are especially important for depth perception of vector field visualization as they provide depth cues. Directional ambient occlusion [61] or ray tracing for tubes [62] is able to create shadow effects for vector field data or field lines extracted from tensor fields (Figures 5(c) and 5(d)).

### 3.3. Tensor Image Visualization

Tensor image visualization techniques concern medical images with the attribute(s) s of equation (1) as tensors—here, we consider only second-order tensors [65], i.e., matrices, and most applications in medicine assume symmetric matrices. DTI captures the diffusion information of water molecules in tissues as symmetric matrices at each point. In medicine, clinical experts often use the term diffusion-weighted imaging (DWI) to refer to the isotropic diffusion map—a scalar measurement of DTI. To avoid confusion, we use the term DTI to refer to the imaging that measures tensor fields and do not use DWI. We classify techniques into three categories for tensor image visualization: *voxel-based*, *glyph-based*, and *tractography*. As in previous sections, we also discuss available techniques for improved spatial perception for tensor medical images. 

#### 3.3.1. Voxel-Based Methods

By computing scalar descriptions of tensors and using 2D slices and/or volume rendering, voxel-based methods convert tensor data visualization to scalar data visualization. One such description is fractional anisotropy [66], and other anisotropy measurements, including linear anisotropy, planar anisotropy, and isotropy are also helpful for describing the shape and size of tensors. Moreover, measurements based on characteristic curves, for example, finite separation ratio, are calculated to describe coherent fiber structures [64]. Tract-based spatial statistics uses nonlinear registration and fractional skeleton to improve the analysis of multisubject DT images [67]. Strategies of voxel-based visualization using direct volume rendering are available, including designing transfer functions based on tensor information and using diffusion volume textures [68].

#### 3.3.2. Glyph-Based Methods

Glyphs allow encoding the complex tensor information using multiple visual channels, for example, shape, size, and color. Classic glyph design uses ellipsoids [69,70] (spherical glyphs) or cuboids that change shape and orientation based on diffusion information—the anisotropy and orientation of principal components. A composite glyph can directly encode linear, planar, and spherical information [71]. Superquadric glyphs [72] with general principles of usage [73] avoid the shortcomings of spherical or cubical glyphs. Uncertainty-aware visualization is available for augmenting spherical, superquadric glyphs for DTI and fourth-order homogeneous polynomial glyphs for high angular resolution diffusion imaging (HARDI) [63]. Aforementioned glyph shapes can be found in Figure 5(a). The spatial arrangement of glyphs is another crucial issue. A glyph packing technique leads to efficient use of space and correct perception of the visualization [74].

#### 3.3.3. Tractography

The main goal of DTI is to reconstruct the white matter fiber tracks of the brain. Tractography extracts fibers or tracts from DTIs and visualizes extracted fibers with vector field visualization techniques. Therefore, tractography is probably the most well-known and popular tensor visualization technique among medical experts. A review on tractography can be found elsewhere [75]. Fiber tracts are typically visualized as lines [57,76] or tubes [77]. GPU acceleration enables interactive visualization of fiber tracks as tubes with tuboids and level-of-detail techniques [78] or hybrid rendering of triangle stripes and point sprites [79].

Visualizing fiber tracts in the context of the scalar medical image [80] helps medical experts better analyze the data. Grouping and clustering fiber tracts reduce clutter and facilitate understanding the anatomy of these tracts. Grouped or clustered tracts are typically color labeled by anatomical bundles [81]. Similar to graph visualization, edge bundling of fiber tracts can also enhance the readability of the visualization [82,83].

#### 3.3.4. Techniques for Improved Spatial Perception

Perception of glyphs is difficult in 3D due to loss of information from projection to the 2D image plane and the ambiguity in the 3D shape representation. There are superquadric glyphs that avoid the ambiguity and improve over spherical and cubical glyphs [72,73]. The glyphs are typically rendered with illuminated surfaces and shadow effects in 3D to further enhance depth perception.

Similarly, perception is an important issue in tractography. Illustrative visualization that provides depth cues helps reduce visual clutter and enhance spatial perception. A survey on illustrative visualization is available elsewhere [84]. Depth-dependent halos [59] enhance depth perception with line-based visualization and depth-based contrast enhancement. Line-based ambient occlusion [85] is devised to enhance depth cues of the tractography visualization in both grayscale and color. Ambient occlusion also enhances depth perception for combined direct volume rendering and fiber tract visualization [61] as shown in Figure 5(b). Shadows could also be generated with ray tracing. In Figure 5(a), a whole brain tractography is visualized by 3D tubes with ray-traced shadows [62]. These techniques complement visualization in context [86] and fiber clustering and bundling [83] for more effective analysis of fiber tracts.

## 4. Medical Visualization Methods for Health Applications

Medical studies on individuals are aimed at providing precise and tailored solutions for the specific anatomy and pathology for individual patients. With the power of medical image visualization, medical experts could deepen the understanding of the data and, therefore, potentially improve the quality of personalized medicine.

Of equal importance is to understand health problems in populations. More specifically, medical image visualization for studies of populations involves ensemble data, i.e., a collection of data members that are individual 3D medical images. Understanding such datasets requires specialized ensemble visualization and visual analysis techniques built on top of basic methods covered in Section 3. Ensemble visualization is aimed at achieving one or more of the following goals: visualizing the main trend of the ensemble, visualizing outliers of the ensemble, and comparing specific members to the main trend or other members. Therefore, we believe that it is necessary and helpful to distinct visualization methods that support medical studies for individuals from those for populations.

In this section, we focus on medical image visualization methods specifically designed to address clinical and public health problems. We cover a broad range of medical visualization methods focusing on medical problems to be tackled (diagnosis, treatment, or prognosis) and with a classification of the scale of the medical study—from individuals to populations.

A summary of papers reviewed in this section can be found in Tables 1– 3 for diagnosis, treatment, and prognosis, respectively. Each paper is documented with the following properties for readers to quickly locate techniques of their interests: supported data types (data type), scale of studies (scale), body locations of the problem (location), and modalities of medical images (modalities). We classify data type into *scalar*, *vector*, and *tensor*; scale is classified into *individual* and *population*; location is defined by the target body part(s) of medical image(s); and modalities show the modality of scanners of medical images.

**Table 1:** Summary of specialized medical image visualization techniques for diagnosis.

Reference	Data type	Scale	Location	Modalities
Lawonn et al. 2016 [87]	Scalar	Individual	Whole body	PET+CT
Jung et al. 2013 [88]	Scalar	Individual	Whole body	PET+CT
Wiemker et al. 2013 [89]	Scalar	Individual	Lymph nodes, lungs, breast, whole body	PET+CT+MR
Termeer et al. 2007 [90]	Scalar	Individual	Heart	Perfusion-MR+MR
Oeltze et al. 2006 [91]	Scalar	Individual	Heart	Perfusion-MR+CTA
Hennemuth et al. 2008 [92]	Scalar	Individual	Heart	MR
Kiri sli et al. 2014 [93]	Scalar	Individual	Heart	CTA+SPECT
Meyer-Spradow et al. 2008 [94]	Scalar	Individual	Heart	SPECT
Williams et al. 2008 [95]	Scalar	Individual	Colon	CT
Mirhosseini et al. 2019 [96]	Scalar	Individual	Colon	CT
Song et al. 2017 [97]	Scalar	Individual	Chest, abdomen	CT
Viola et al. 2008 [98]	Scalar	Individual	Liver	US+CT
Zhou and Hansen 2014 [34]	Scalar	Individual	Brain	MR
Jösson et al. 2020 [99]	Scalar	Population	Brain	fMR+MR
Elbaz et al. 2014 [53]	Vector	Individual	Heart	PC-MR
Meuschke et al. 2016 [100]	Vector	Individual	Heart	PC-MR
Köhler et al. 2013 [52]	Vector	Individual	Heart	PC-MR
Born et al. 2013 [55]	Vector	Individual	Heart	PC-MR
van Pelt et al. 2010 [101]	Vector	Individual	Heart	PC-MR
van Pelt et al. 2011 [49]	Vector	Individual	Heart	PC-MR
Zhang et al. 2016 [102]	Tensor	Population	Brain	DT
Zhang et al. 2017 [103]	Tensor	Population	Brain	DT

**Table 2:** Summary of specialized medical image visualization techniques for treatment.

Reference	Data type	Scale	Location	Modalities
Rieder et al. 2008 [104]	Scalar	Individual	Brain	MR
Weiler et al. 2011 [105]	Scalar	Individual	Brain	MR
Khlebnikov et al. 2011 [106]	Scalar	Individual	Abdomen	CT
Beyer et al. 2007 [107]	Scalar	Individual	Brain	MR
Dick et al. 2011 [108]	Scalar	Individual	Bone	CT
Lundstrom et al. 2011 [109]	Scalar	Individual	Bone	CT
Smit et al. 2007 [110]	Scalar	Individual	Pelvic	MR
Butson et al. 2013 [111]	Scalar	Individual	Brain	MR
Vorwerk et al. 2020 [112]	Scalar	Individual	Brain	MR
Bock et al. 2013 [113]	Scalar	Individual	Brain	MR
Athwale et al. 2019 [114]	Scalar	Individual	Brain	MR
Blaas et al. 2007 [115]	Tensor	Individual	Brain	fMR+MR+DT
Born et al. 2009 [116]	Tensor	Individual	Brain	fMR+MR+DT
Diepenbrock et al. 2011 [117]	Tensor	Individual	Brain	fMR+MR+DT
Joshi et al. 2008 [118]	Tensor	Individual	Brain	fMR+MR+DT
Rieder et al. 2008 [119]	Tensor	Individual	Brain	fMR+MR+DT
Dick et al. 2009 [120]	Tensor	Individual	Bone	CT+sim

Note: sim: simulation.

**Table 3:** Summary of specialized medical image visualization techniques for prognosis.

Reference	Data type	Scale	Location	Modalities
Raidou et al. 2016 [127]	Scalar	Population	Prostate	MR
Karall et al. 2018 [128]	Scalar	Population	Breast	MR
Raidou et al. 2018 [129]	Scalar	Population	Bladder	CT
Furmanová et al. 2021 [130]	Scalar	Population	Prostate, bladder, rectum	CT

### 4.1. Diagnosis

Methods designed for diagnosis are summarized in Table 1. While the majority of these methods handle studies on the individual level, a few recent works support studies of populations. 

#### 4.1.1. Individual

Diagnosis for individuals often requires multimodal medical images to provide sufficient information. In oncology, positron emission tomography (PET) images that show physiological functions and CT images that represent anatomical structures are used jointly for the diagnosis of tumors. Multimodal visualization techniques are, therefore, required to analyze the combination of the two types of scans. Focusing only on potential PET anomaly regions with the CT anatomy as the context, i.e., focus-and-context visualization, is an effective visualization strategy for PET+CT images. An illustrative technique allows us to visualize the CT data as the context in cartoon style and the PET data as focus with a see-through lens that quickly draws the attention of medical experts [87]. There, the focus can be interactively manipulated and contents within focal regions are controlled by interactive transfer functions. Alternatively, a visibility-based transfer function for PET+CT data allows users to select regions of interest for further analysis [88]. Shape-encoded rendering combines shape analysis with volume rendering to highlight tubular and nodular structures [89]. The method aids the diagnosis of anomalies in CT scans of lungs or PET/CT scans for oncological practices.

In the field of cardiology diagnosis, perfusion data from MR or single photon emission computed tomography (SPECT) are used in conjunction with the regular MR or CT images that are of higher resolutions to indicate the underlying anatomy. The analysis of coronary artery disease is achieved by integrating the perfusion MR with the morphologic data from CT angiography (CTA) using visual analysis with multiple-linked views [91]. A well-accepted analysis tool in cardiac diagnosis, the bull’s eye plot is extended for rest and stress comparison and is used interactively to drive the 3D exploration with colored height fields, icons, and synchronized lenses. The bull’s eye plot is further extended to be continuous and as a volume to assess transmurality in a 3D anatomical context [90]. Diagnosis is achieved with visual analysis supported by a comprehensive visualization and interactive exploration with multiple-linked views, and several segmented volumes and enhanced MR images are used for the joint rendering. A multivariate glyph-based method enables the structured analysis of myocardial perfusion using 3D glyphs encoding parameters of the left ventricular myocardium [94]. By linking the 3D view with 2D slices, the method supports the analysis of normal case, various types of ischemia, and heart failure. CTA and perfusion SPECT images are combined and jointly analyzed to diagnose coronary artery disease [93]. A study comparing the method to the traditional practice shows that the visualization method is advantageous in terms of diagnostic performance. Contrast-enhanced cardiac images, including perfusion images, whole-heart coronary angiography, and late enhanced images, are analyzed by aligning different datasets together and visualized as multiple isosurfaces [92].

Another important medical image visualization-based diagnosis approach is virtual endoscope visualization, e.g., virtual colonoscopy and gastroscopy, where the visualization of inner surfaces of tubular structures is the main focus. For example, an immersive virtual colonoscopy method supports the exploration of the colon within volume visualization in a virtual reality environment [96]. A hybrid technique that combines the inner surface rendering and volume rendering of colons is available [95]. For a full review of flattening visualization techniques, we refer readers to a survey elsewhere [7].

Extensive training is required for diagnosis with medical images. A visual analysis method enables comparative visualization of gaze data of several radiologists reading slices and volume rendering of medical images [97]. By setting in a real diagnostic environment, the method is useful for training radiologists. Ultrasound (US) images are frequently used in clinical practice, but effective diagnosis with such images requires extensive training. A joint 2D US and 3D CT image visualization method registers the 2D plane of the US image to segmented 3D structures of CT to assist the learning of liver examinations [98].

The 4D phase-contrast MR (PC-MR) is a recent advancement in medical imaging that is designed for measuring time-varying flow fields in the body, which is specifically used for hemodynamics analysis. Among other features, the vortex is especially useful in the analysis and diagnosis of cardiac flow data. Vortex rings in the left ventricle are extracted and visualized in 3D to analyze inflow during early and late diastolic filling of normal subjects [53]. Quantitative parameters characterizing vortex flow for these phases are formulated for normal subjects. Aortic vortex flow is classified based on the orientation, shape, and temporal occurrence of the vortex for PC-MR data of healthy subjects and ones with cardiovascular diseases [100]. The classification results are visualized with 2D vortex plots and 3D glyph visualization.

The flow field in the heart and aorta is analyzed by semiautomatic segmentation with line predicates that extract vortices and visualized with arrows [52]. The most suitable cardiac blood flow vortex extraction criterion is found through comparison, investigating pathologies like coarctations, tetralogy of Fallot, and aneurysms. A visual analysis method provides flexible interactive exploration of cardiac blood flow using line predicates that generate bundles with similar flow characteristics [55]. The technique can be applied to healthy and pathological hearts and shows aspects of flow that cannot be seen with traditional methods.

#### 4.1.2. Population

Diagnosis can benefit from studying health problems in a population, for example, with a cohort study, and by comparing different individuals. Traditionally, cohort studies with medical images rely on hypothesis formation and statistical analysis, but the visualization and exploration of the imaging data are ignored. A visual analysis method combines hypothesis formation and reasoning with interactive volume rendering of multivariate brain MRI and fMRI cohort study data [99]. With multiple-linked views, the method supports the exploration of the bidirectional correlations between the volume rendering and clinical parameters and the comparison of different patient groups.

Diagnosis can be potentially further improved by including tensor information of DTIs. However, visualization of DTIs in a population is challenging because, on top of the occlusion issue of spatially overlapping images, each voxel there encodes complex information. As a first step, effective comparative visualization of two DTI images is required. A glyph-based technique visualizes three aspects of tensors, namely, the scale, the anisotropy type, and the orientation [102]. By showing the glyphs on 2D slices, the method is able to compare two DTI images. As an example, the brain DTI of a healthy subject is compared to an HIV-infected subject. An overview+detail visualization is devised for DTI image ensembles: aggregate tensor glyphs show an overview of the ensemble in the spatial layout, and visualizations of tensor properties (scale, shape, and orientation) are used for detailed analysis [103]. A case study demonstrates that the method is able to visualize and analyze a cohort DTI study of 46 subjects.

### 4.2. Treatment

Treatments aided by medical visualization are mainly surgical planning and therapeutical intervention planning. Therefore, treatment-related visualization methods are individual-based as shown in Table 2. A thorough introduction of various applications of visualization in surgical planning can be found elsewhere [121]. 

Neurosurgery preoperative planning is a major task for visualization techniques for brain imaging. Due to the complexity and importance of the brain, multimodal 3D medical images are used in neurosurgery to locate different anatomical structures. Heterogeneous pathological tissues are visualized with volume rendering by registering multimodal volumes, e.g., T1, T2, and FLAIR MRI, and automatically segmented mask volumes [104]. A slice-based interface that is familiar to medical experts is used to drive the visual exploration of multimodal brain images with direct manipulation on the 2D images with lassos [34]. Transfer functions are semiautomatically designed based on user-selected 2D regions, and then, brain tumors and edema can be visually segmented in 3D and visualized with volume rendering. Vascular structures in the brain are extracted and visualized with volume rendering to aid neurosurgery planning for arteriovenous malformations [105]. Here, feeding arteries, draining veins, and arteries “en passage” are segmented and visualized together with the brain rendered as the context. A high-quality multimodal scalar volume visualization method facilitates the actual planning of neurosurgeries [107]. The method supports specific operation planning, for example, the optimal skin incision and skull opening for the pathology and customized surgery of deep-seated lesions for a given patient; specialized visualization of superficial brain anatomy, function, and metabolism facilitates the planning. 

Using DT images jointly with fMRI and regular MR scans could further improve the quality of surgical planning as fiber tracts and functional regions of the brain around the tumor could be analyzed. DTI, fMRI, and regular MRIs are combined and visualized with volume rendering and tube-based rendering for brain tumor resection planning [115]. Fiber bundles can be interactively selected so that those around the tumor could be avoided in the planning. The fMRI activation areas, functional areas of the brain, and fiber tracts connecting these areas are jointly visualized with illustrative rendering [116]. Interactive probing of fMRI, DTI, and MRIs within the brain visualization is proposed for neurosurgical planning [117]. Uncertainty of these images is also visualized in the method to provide additional information to the user. Interactions, especially, cropping or cutting operations from the surface of the brain to the inside, are critical for neurosurgery planning. Volume clipping with complex geometries [122] is the foundation for such tailored cropping operations. Cropping views with different shapes, e.g., sphere, cube, and cylinder, are combined with an image-guided navigation system that visualizes MRI, fMRI, DTI, and SPECT for epilepsy neurosurgery [118]. Distance information is critical in preoperative planning, and DTI and fMRI provide such data for fiber tracts and functional regions. In a comprehensive method designed for neurosurgical planning, the tumor and neighboring fiber tracts are rendered as the focus with distance-based enhancements while the volume-rendered brain provides the context [119]. The planned path can be interactively manipulated and is visualized as a line and as a cylindrical cropping window on the brain. 

In an oncology surgery, multiple possible paths to a tumor may exist. However, the safety of paths is not equal and has to be considered during the planning. A ray-based method estimates the safety of all straight access paths to the tumor in volume rendering and provides the area and path safety information [106]. Clear evidence shows that the method is liked by medical experts and can be used in clinical practice with little overhead. Pelvic oncology surgery planning is aided with a visual analysis method based on preoperative MR scans [110]. The method is built on an atlas by registering the MRIs of a patient to visualize the context (organs around the tumor), the target (the tumor), and risks (autonomic nerves) of the surgery. Distances between nerves to the mesorectum and the tumor to the mesorectum, which are critical to the surgery, are calculated as a distance field. A linked-view tool comprising a 3D model view, an MRI view, and a distance field-based unfolded view is implemented. Five medical experts evaluated the method and considered that it has potential in surgical planning and surgical training for oncologic surgeons. 

Precise preoperative planning is also critical in orthopedics. A medical visualization table is available to visualize CT scans using volume rendering with user interface and interactions designed for low learning effort and similarity to the real working scenarios for surgeons [109]. A user study shows that the table system is liked by surgeons and potentially beneficial for planning. Hip joint replacement is an important surgery in orthopedics. The optimal implant positioning can be aided by an interactive distance field visualization technique that uses glyphs and slices in a 3D isosurface context to show distances between the implant and the bone boundaries [108]. Stress simulation is an effective method for the design and the planning of the implant; however, the resulting stress tensor fields need special visualization methods as most methods are designed for diffusion tensors. With volume rendering and line rendering, a focus-and-context method is proposed to visualize time-varying stress tensor fields generated by such simulations [120]. The method supports the interactive exploration of the simulation and reacts to changes in the simulation and therefore could compare the physiological stress distribution before and after the simulated replacement surgery. 

Deep brain stimulation (DBS) is an accepted neuromodulation therapy for treating the motor symptoms of Parkinson’s disease. The DBS device that generates electrical stimulation as an alternation of neural activity is comprised of a multielectrode lead implanted in the brain and a connected subcutaneous implantable pulse generator. The accuracy of the multielectrode lead placement is the key to the effectiveness of the therapy. A mobile device-based visualization tool supports volume rendering and isosurface rendering to compare different settings of DBS and help healthcare providers to choose the optimal configuration for a patient [111]. A further improvement uses a client-server approach to achieve efficient interactive visualization and simulation of DBS [112]. The usefulness of the method is demonstrated by a postoperative example and another example of DBS surgery pre- and interoperative planning. The precision of DBS electrode positioning is related to the uncertainty introduced by the resolution of brain imaging. The positional uncertainty of each electrode is quantified and visualized with uncertainty-aware volume and isosurface visualization techniques [114]. Multimodal volumes and their associated uncertainty are quantified and fused in a multiview visual analysis method to assist the planning of DBS electrodes [113]. This comprehensive method covers the planning, recording, and placement phases of the treatment and uses volume and geometry rendering, spatiotemporal visualization, and uncertainty visualization for corresponding elements in the procedure. 

A relevant topic that requires high-quality visualization is the segmentation of 3D anatomy and lesions from medical images. Segmentation is often necessary for the diagnosis and treatment of individuals and, therefore, is a prerequisite for many of the aforementioned visualization techniques. Accurate and efficient segmentation of specific anatomical structures, for example, the heart [123, 124] and the prostate [125], has been an active and long-standing research area in medical imaging. A discussion of state-of-the-art segmentation techniques is beyond the scope of our paper and can be found elsewhere [126]. In Section 6, we list free visualization software dedicated to 3D segmentation. 

### 4.3. Prognosis

In contrast to the case of treatment, the value of visualization lies in its capability of comparison in a population to aid the prognosis evaluation. Therefore, all techniques for prognosis summarized in Table 3 are population-based. A number of techniques focus on radiotherapy treatment evaluation. A visualization method is available for exploring and understanding tumor control probability models for cohorts [127]. By combining visualizations of medical images and statistical models, the method supports the exploration of uncertainty, parameter sensitivity analysis, interpatient response variability identification, and finding treatment strategies that result in the desired outcome. 

A cohort study of patients who underwent breast cancer chemotherapy treatment is analyzed with a visualization method that shows different aspects of the study using multiple-linked views [128]. The method combines medical images and nonimage information in an interactive visualization tool that allows for analyzing individual patients, comparing different chemotherapy treatment strategies and comparing different patients. 

Radiotherapy-induced bladder toxicity is analyzed with a visualization technique tailored to investigating individual patients and cohorts in the whole treatment process of a cohort study [129]. This method focuses on the analysis of the impact of shape variations on the accuracy of dose delivery by integrating the spatial visualization of bladders, dimensionality reduction and clustering, and dose distribution visualizations. The idea is further extended to visualize and analyze more organs that may impact the accuracy of dose delivery in radiotherapy treatment for prostate cancer [130], and its usefulness has been demonstrated through the exploration of cohort studies by health experts. 

## 5. Future Directions and Limitations of Medical Image Visualization

Some visualization methods support exploring and analyzing medical image data from basic medical research that can potentially address unsolved health challenges in the future. In this section, we discuss some of these important future directions and also limitations of medical image visualization. In Table 4, we summarize medical image visualization techniques with potential health science applications. Here, we list the features of these methods as a reminder to the readers of what are available in the visualization tool box in the future.

**Table 4:** Techniques with potential health science applications.

Reference	Scale	Location	Modalities	Features
Zachow et al. 2009 [131]	Individual	Nose	CT+sim	Fluid dynamics: speed, pressure, humidity, temperature
Gasteiger et al. 2012 [56]	Individual	Brain	CTA+sim	Fluid dynamics: inflow jet and impingement zone
Meuschke et al. 2019 [132]	Individual	Brain	CTA+sim	Fluid dynamics: vortex, blood flows
Rosen et al. 2016 [133]	Individual	Heart	MR+DT+sim	Bioelectric fields
Meuschke et al. 2017 [134]	Individual	Brain	Sim	Rupture risk of aneurysms, stress tensor
Zhou et al. 2021 [135]	Population	Brain, heart	MR+sim	Quantitative comparison of scalar medical images

Note: sim: simulation.

Simulations are an important approach in medical research to understand complex, invisible, and/or perpetual activities in human bodies. Nasal flow simulation is an important means for understanding the physiological nasal breathing that improves the overall traditional statistics-based summary of flow behaviors. A visual analysis method aids in the exploration of a computational fluid dynamics simulation on an anatomically correct model of the upper respiratory tract [131]. With multiple-linked views, the method allows users to analyze multiple attributes, e.g., speed, pressure, humidity, and temperature, of the complex flow simulation to derive intervention plans. Hemodynamic characteristics in cerebral aneurysms are studied with computational fluid dynamic simulations and segmented data from CTA scans [56]. The visual analysis tries to understand the inflow jet and impingement zone that are correlated with the risk of rupture. A vortex classification method automatically classifies blood flows in cerebral aneurysms and visualizes the clusters as streamlines in an aneurysm-based and a hemisphere-based visualization [132].

Cardiac diseases are typically related to malfunctions in bioelectric fields in the body that are difficult to measure in vivo. Simulation of such bioelectric fields then becomes a feasible alternative, and, therefore, in-depth analysis of simulation results is important. Early work focuses on the visualization of torso electric field simulations [136]. Specialized for the 3D myocardial ischemia simulation, a multiple-linked view approach is devised to perform the visual analysis of the multiple simulation runs of bioelectric fields on the heart [133], which could also potentially be used for diagnosis. A systematic discussion of computational and numerical methods for bioelectric fields problems can be found in a review, where related visualization techniques are also discussed [44].

Comparative visualization is important for understanding an ensemble of simulation runs and comparing between patients or different measures of medical images. Scalar image ensembles, for example, brain MR atlas data (https://www.oasis-brains.org/), are a common form of ensemble medical image data. Direct visualization of image members in 2D and 3D is not effective due to occlusion, and quantitative comparison is not feasible in this way either. One possible solution is to reduce the dimensionality of image data to one (1D) with space-filing curves [135, 137, 138]. Data-driven space-filling curves [135] better preserve spatial coherency in the resulting 1D representation than static curves [137, 138], which potentially break spatially coherent features into distant 1D fragments. The method is applied for visualizing brain MRI atlas and an ensemble of 3D myocardial ischemia simulations and could potentially be used for diagnosis or research by finding anomalies of subjects through comparison to the main trends. Comparative visualization of stress tensors is available for analyzing rupture risks of cerebral aneurysms based on computational fluid dynamic simulations [134]. With several glyph designs, the method supports the comparison of local stress tensors on the inner and outer vessel walls. Medical experts consider that this method introduces the often overlooked wall structure information for rupture assessment and could contribute to the development of a comprehensive risk factor of aneurysms in the future.

Medical image visualization has its limitations. First, customized techniques and tools are required for specific medical problems, which demand close collaborations between the visualization and medical experts. Typically, an iterative process with several prototypes is required for a method and its associated software tool to become usable, which is often time-consuming. Second, a learning process is required for medical experts to familiarize themselves with new concepts or interactions, for example, transfer function design in volume rendering. Nevertheless, medical image visualization has the advantage of combining the expertise of humans and the computational power of machines, which is vital for health science, making it a promising research direction.

## 6. Software Tools for Medical Image Visualization

Both commercial tools and free software are available for medical image visualization. In this paper, we list representative free software that can be found on the Internet and readily used for 3D medical images of various types and file formats. As shown in Table 5, we briefly summarize these tools with data types that can be handled (data types) as well as their featuring characteristics (features).

**Table 5:** Medical image visualization tools.

Name	Data types	Features
ParaView	Scalar, vector, tensor	Analysis, large datasets, parallel/super computing
Voreen	Scalar, vector	Rapid prototype
Inviwo	Scalar, vector	Rapid prototype
MegaMol	Scalar, vector	Particles, rapid prototype
SCIRun	Scalar, vector, tensor	Modeling, simulation, analysis
3D Slicer	Scalar	AI, segmentation
Seg3D	Scalar	Segmentation
ImageVis3D	Scalar	Large datasets
FluoRenderer	Scalar	Confocal microscopy data

Visualization and analysis tools for general scientific problems and data provide flexible rapid prototyping frameworks. ParaView (https://www.paraview.org/) is a cross-platform open-source visualization tool designed for interactive visualization and data analysis for a wide range of research and engineering areas, and various types of medical images (scalar, vector, and tensor) are supported [139]. Voreen (voreen.uni-muenster.de) is an open-source rapid application development framework for the interactive visualization and analysis of multimodal volumetric datasets [140]. Inviwo (https://inviwo.org/) is a framework for rapid prototyping visualizations and provides a rich visual interface for creating customized visualization [141]. MegaMol (https://megamol.org/) is a comprehensive cross-platform visualization prototyping framework evolved from particle rendering for molecular datasets [142, 143]. SCIRun (https://www.sci.utah.edu/software/scirun.html) is a software environment for scientific problem simulation, modeling, and visualization, and it supports various types of medical images.

A number of tools are available for scalar medical image visualization and analysis. 3D Slicer (https://download.slicer.org/) is a tool for visualization and analysis of medical images and features interface for medical devices, for example, surgical navigation system and robotic devices [144]. Seg3D (https://www.sci.utah.edu/software/seg3d.html) is a medical volume segmentation and processing tool that allows for flexible manual segmentation and a number of automatic segmentation algorithms. ImageVis3D (https://www.sci.utah.edu/software/imagevis3d.html) is a scalable and multiplatform volume visualization tool that supports large datasets and works on mobile devices [145]. FluoRenderer (https://www.sci.utah.edu/software/fluorender.html) features the visualization of confocal microscopy data and multichannel scalar volume datasets.

These software tools contain example datasets, tutorials, detailed user guides, and supporting communities. Readers are encouraged to try out these tools with included datasets and their own medical images to have first-hand experience of some key techniques discussed in this paper.

## 7. Conclusion

In this paper, we have provided an overview of 3D medical image visualization techniques. Starting from a classification of medical images in terms of data characteristic, i.e., the mathematical properties of field data, we review fundamental visualization techniques for scalar, vector, and tensor medical images. The discussion covers seminal work that lays foundations for each type of visualization and features techniques that allows for accurate spatial perception that is important in medical practices. Next, specialized medical visualization techniques are categorized based on the supported data type, medical procedures, the scale of the concerned medical problems, locations of the studied problems, and modalities of images. We describe the medical aspects as well as technical aspects of each technique to facilitate medical experts to choose proper techniques for their specific problems. Then, we discuss works that have potential health science applications and the limitations of medical image visualization. Finally, state-of-the-art free visualization software is listed so that medical experts can have a first-hand experience of some of the aforementioned techniques and experiment with their own data to gain insights into visualization.
